# Astragalus Extract Mixture HT042 Reverses Cyclophosphamide-Induced Immunosuppression Through Dual Modulation of Innate and Adaptive Immunity

**DOI:** 10.3390/ijms26104850

**Published:** 2025-05-19

**Authors:** Se-Young Kim, Joohee Son, Minju Kim, Chae Yun Baek, Mi-Yeon Kim, Ari Shin, Donghun Lee, Hocheol Kim

**Affiliations:** 1 Korea Institute of Science and Technology for Eastern Medicine (KISTEM) NeuMed Inc., 88 Imun-ro, Dongdaemun-gu, Seoul 02440, Republic of Korea; tpdud74@neumed.co.kr (S.-Y.K.); zoe_ju@neumed.co.kr (M.K.); okkite@neumed.co.kr (M.-Y.K.); 2 Department of Herbal Pharmacology, College of Korean Medicine, Gachon University, 1342 Seongnamdae-ro, Sujeong-gu, Seongnam-si 13120, Republic of Korea; sonju0222@gachon.ac.kr (J.S.); cyning20@gachon.ac.kr (C.Y.B.); 3 Department of Herbal Pharmacology, College of Korean Medicine, Kyung Hee University, 26 Kyungheedae-ro, Dongdaemun-gu, Seoul 02447, Republic of Korea; ari1112@naver.com

**Keywords:** HT042, immunomodulation, cyclophosphamide, immunosuppressed mice, innate immunity, adaptive immunity, T cell activation

## Abstract

Deficiencies in immune function increase susceptibility to infections and chronic diseases by impairing immune surveillance and tolerance mechanisms, especially in children with immature immune systems. Chronic inflammation associated with immune dysfunction can impair childhood by suppressing the GH–IGF-1. HT042 is composed of *Astragalus mongholicus*, *Eleutherococcus senticosus*, and *Phlomis umbrosa*, which are medicinal herbs that are traditionally utilized in East Asia to promote growth and enhance immune function; thus, HT042 itself holds potential as an immunomodulator. We evaluated the immunomodulatory effects of HT042 in a cyclophosphamide (CYP)-induced immunosuppressed mouse model, as well as in ex vivo primary splenocytes and RAW 264.7 macrophages. HT042 demonstrated remarkable immune-enhancing effects, including the restoration of weight loss and hematological parameters, as well as enhancing NK cell activity. Primary splenocytes treated with HT042 showed increased expression of CD3, CD4, and CD8, along with Th subset transcription factors (T-bet, GATA3, RORγt, Foxp3) and corresponding cytokines (IFN-γ, IL-4, IL-17, IL-10). In RAW 264.7 macrophages, HT042 increased nitric oxide production and upregulated NOS2, COX-2, and inflammatory cytokines (IL-6, IL-1β, TNF-α). It is noteworthy that HT042 enhances both innate and adaptive immune pathways, particularly via T cell modulation and macrophage activation, as this study is among the first to demonstrate such effects in the context of CYP-induced immunosuppression.

## 1. Introduction

Deficiencies in immune function predispose individuals to a heightened risk of infection and may contribute to the chronic progression and recurrence of diseases such as recurrent pneumonia, tuberculosis, and herpes zoster [1]. They also impair tumor immune surveillance and disrupt regulatory mechanisms that maintain immune tolerance. The innate immune system acts as the front line of defense against invading pathogens by rapidly mobilizing key effector cells (e.g., neutrophils, natural killer (NK) cells, and macrophages) and cytokines, thereby identifying and eliminating threats in a non-specific manner. The adaptive immune system, primarily mediated by T and B lymphocytes, complements innate immunity by specifically recognizing non-self-antigens, initiating clonal expansion and antigen-specific responses, and facilitating the precise elimination of pathogens and infected cells [2].

In recent years, the incidence of secondary immunodeficiency has risen due to various environmental and lifestyle-related factors such as chronic stress, sleep deprivation, excessive antibiotic use, high-fat and high-sugar diets, and the widespread consumption of processed food [3]. Children are particularly susceptible to immune challenges due to their underdeveloped immune systems. They display diminished phagocytosis, impaired antigen presentation, weakened pattern recognition receptor (PRR) signaling and limited memory T and B cell repertoire, leading to suboptimal adaptive immune responses against pathogens [4]. Frequent infections and immune dysfunction in early childhood can lead to transient growth delays, primarily through the action of pro-inflammatory cytokines which suppress the growth hormone (GH)–insulin-like growth factor-1 (IGF-1) axis [5]. In recent years, immunomodulatory strategies using micronutrients (e.g., vitamin D_3_, omega-3 fatty acids, zinc) and natural products (e.g., yeast-derived β-glucan, ginseng, and medicinal mushrooms) have emerged as promising options for the long-term management of complex diseases including infections, cancers, and autoimmune disorders [6,7]. Natural-product–based immunomodulators offer therapeutic promise due to the synergistic interactions among their constituents and the presence of multiple bioactive compounds that act on diverse targets involved in disease pathophysiology [8]. Furthermore, their long-standing use in traditional medicine, supported by empirical evidence, adds to their perceived safety and tolerability.

HT042 is the first functional ingredient globally to be clinically validated for its efficacy in promoting height growth. In 2014, it was approved by the Korean Ministry of Food and Drug Safety (MFDS) as a functional food ingredient that supports height development in children. The efficacy of HT042 in promoting height growth has been validated in two clinical trials, with one study reporting more pronounced effects in the third month compared to the initial two months. HT042 is a standardized herbal formulation consisting of *Astragalus mongholicus* Bunge, *Eleutherococcus senticosus* (Rupr. & Maxim.) Maxim., and *Phlomis umbrosa* (Turcz.) Kamelin & Makhm., which are medicinal herbs traditionally used in East Asia that have been extensively studied for their anticancer and immunomodulatory properties. Polysaccharides from *Astragalus mongholicus* Bunge and *Eleutherococcus senticosus* (Rupr. & Maxim.) Maxim. have been shown to modulate both innate and adaptive immunity by enhancing macrophage, NK cell, and T cell activity through cytokine regulation and gut microbiota modulation [9,10,11]. It is noteworthy that HT042 enhances GH secretion, which may in turn promote systemic and local IGF-1 synthesis. IGF-1 serves as a key endocrine mediator of physiological growth and contributes to immune regulation. Disruption of the GH–IGF-1 axis during childhood has been linked to both growth retardation and immune dysfunction [12]. GH and IGF-1 enhance immune recovery by promoting hematopoiesis and facilitating the differentiation and activation of immune cells—including T and B lymphocytes, PBMCs, and NK cells—primarily through the JAK2/STAT5, PI3K/Akt, and NF-κB signaling pathways [13,14,15]. Based on these findings, it is plausible that HT042 supports immune recovery by modulating the GH–IGF-1 axis. This is further supported by the results of previous studies demonstrating that HT042 restores the expression of IGF-1R and GHR suppressed by the immunosuppressant dexamethasone (DEX) [16].

This study evaluated the immune-enhancing potential of HT042 in a cyclophosphamide (CYP)-induced-immunosuppression mouse model, complemented by in vitro assays. CYP is an alkylating agent that interferes with DNA replication, and it is widely utilized as a chemotherapeutic agent and immunosuppressant [17]. We examined whether HT042 could restore innate and adaptive immune cell populations and regulate immunomodulatory cytokines, with particular emphasis on its effects on T lymphocytes.

## 2. Results

### 2.1. HPLC Chromatograms of HT042

**Figure 1:**
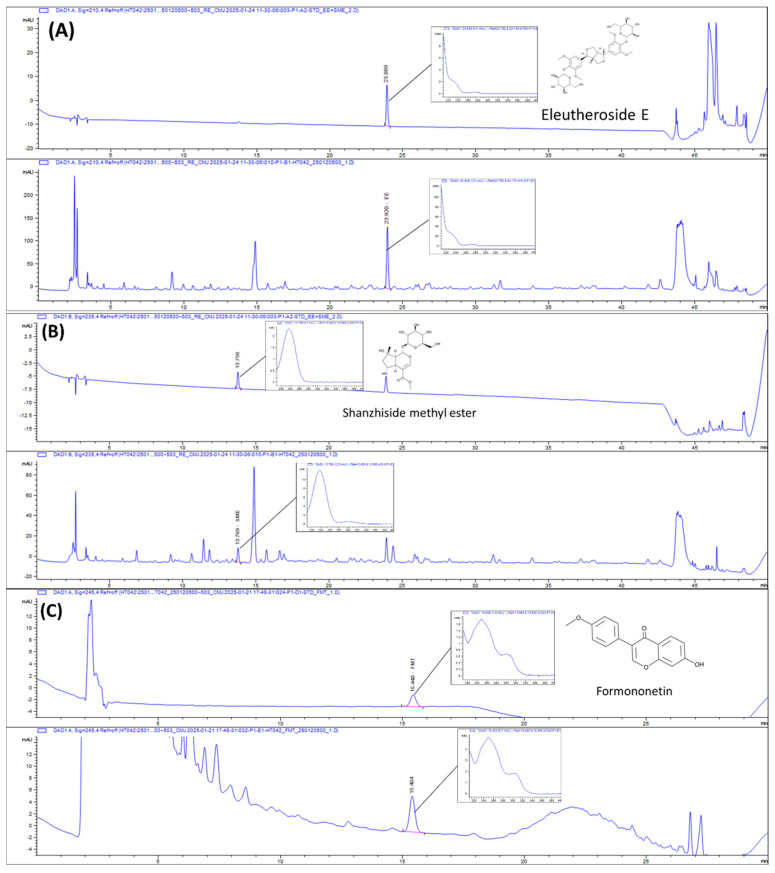
HPLC chromatograms of eleutheroside E (**A**), shanzhiside methyl ester (**B**), and formononetin (**C**) in HT042. Upper panels show chromatograms of standard solutions; lower panels show those of the HT042 extract.

The concentrations of formononetin, eleutheroside E, and shanzhiside methyl ester in HT042 were estimated using HPLC analysis (Figure 1). HT042 contains 3.60 ± 0.024 mg/g of eleutheroside E, 1.75 ± 0.014 mg/g of shanzhiside methyl ester, and 0.08 ± 0.001 mg/g of formononetin.

### 2.2. Effects of HT042 on the Body Weight of Mice with CYP-Induced Immunosuppression 

**Figure 2:**
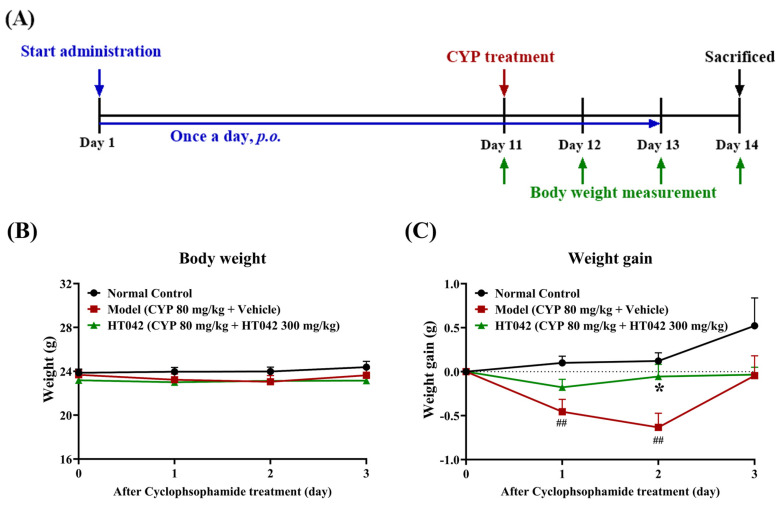
Effects of HT042 on the body weight of mice with CYP-induced immunosuppression. (**A**) In vivo experimental design. BALB/c mice were randomly distributed into three groups. Mice in the HT042 group received oral administration of HT042 300 mg/kg/day for 10 days prior to CYP treatment. Distilled water was used as a vehicle. On Day 11, CYP was administered intraperitoneally at a dose of 80 mg/kg to induce immunosuppression. (**B**) Body weight measurements and (**C**) weight gains following CYP administration. All data are shown as mean ± SEM. ##, *p* < 0.01 and *, *p* < 0.05 represent a significant difference between the Model and the HT042 groups by two-way ANOVA and Dunnett’ s test. NC: normal control; CYP: cyclophosphamide.

Mice in the HT042 group received oral HT042 for 10 days prior to CYP administration. On Day 11, CYP was intraperitoneally injected at a dose of 80 mg/kg into both the Model and HT042 groups to induce immunosuppression. Body weight was monitored daily from Day 11 to Day 14 (Figure 2A). Body weight did not differ among the groups on Day 11. However, from Day 12, both CYP-treated groups exhibited weight loss, with no significant difference in the extent of reduction at that time point (Figure 2B). Notably, the HT042 group showed a significantly smaller decrease in body weight on Day 13 compared to the Model group. While the Model group began recovering weight on Day 14, the HT042 group exhibited only minimal weight loss on Day 12 and returned to baseline levels by Day 13, indicating a more rapid recovery (Figure 2B).

### 2.3. Effects of HT042 on the Proliferation of Primary Splenocytes

**Figure 3:**
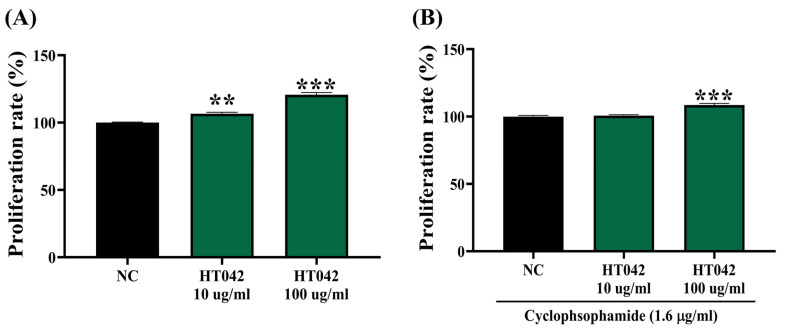
Effects of HT042 on splenocyte proliferation. (**A**) Splenocyte proliferation treated with HT042 under normal conditions. (**B**) Splenocyte proliferation treated with HT042 under CYP induced-immunosuppressive conditions. Data represent the percentage of cell proliferation relative to the optical density (OD) value of the negative control (NC). All data are shown as mean ± SEM. Statistical significance relative to the NC group is indicated by ** *p* < 0.01 and *** *p* < 0.001. NC: normal control.

The effect of HT042 on the proliferation of primary splenocytes was evaluated in mice under two experimental conditions: a normal immune state and an immunocompromised state induced by cyclophosphamide (CYP) treatment, which promotes immune cell apoptosis (Figure 3). After 24 h of incubation, results showed that under normal immune conditions, HT042 significantly enhanced splenocyte proliferation in a dose-dependent manner with an approximate 20.7% increase observed at 100 μg/mL compared to the normal control (Figure 3A). A comparable proliferative effect was also observed under cyclophosphamide-induced immunosuppression, with an 8.6% increase relative to the normal control group (Figure 3B). These results indicate that HT042 promotes the proliferation of primary splenocytes, even under immunocompromised conditions.

### 2.4. Effects of HT042 on the Hematocytes of Mice with CYP-Induced Immunosuppression 

**Figure 4:**
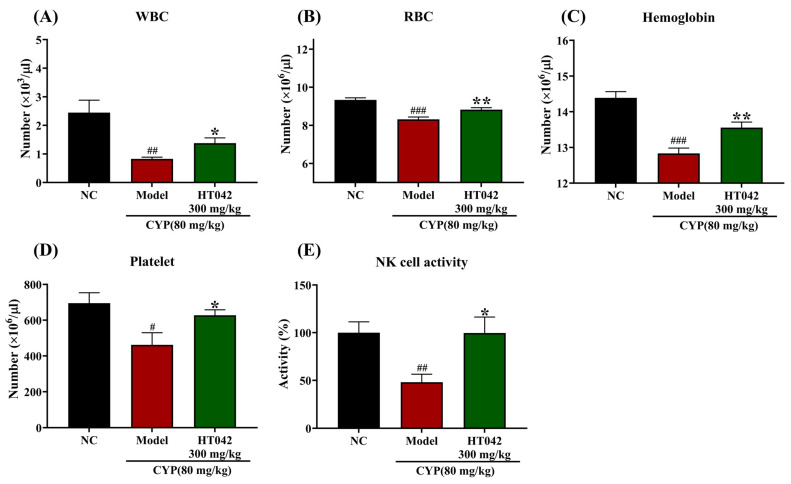
Effects of HT042 on hematocytes of mice with CYP-induced immunosuppression. Mice in the HT042 group received oral administration of HT042 (300 mg/kg/day) for 10 days prior to CYP treatment. Distilled water was used as a vehicle. CYP (80 mg/kg) was injected intraperitoneally to induce immunosuppression. CYP 80 mg/kg was administered intraperitoneally to induce immunosuppression. (**A**) White blood cell (WBC) count, (**B**) red blood cell (RBC) count, (**C**) hemoglobin level, and (**D**) platelet count, (**E**) NK cell activity represented by IFN-γ secretion levels, expressed as a percentage relative to the NC group. All data are shown as the mean ± SEM. * *p* < 0.05 and ** *p* < 0.01 differences between Model and HT042, and # *p* < 0.05, ## *p* < 0.01, and ### *p* < 0.001 differences between NC and Model groups. NC: normal control; CYP: cyclophosphamide.

To evaluate the hematopoietic effect of HT042, we estimated the numbers of white blood cells (WBCs) and the activity of NK cells, as well as hematological parameters including red blood cells (RBCs), hemoglobin, and platelets in CYP-induced mice. CYP treatment significantly reduced WBC, RBC, hemoglobin, and platelet counts when compared to the NC group. In contrast, the HT042 group significantly recovered WBC, RBC, hemoglobin, and platelet counts (Figure 4A–D). A significant decline in NK cell activity, which was indicated by IFN-γ secretion, was observed in the Model group by approximately 52.0% compared to the NC group. The HT042 group exhibited a significant elevation in IFN-γ secretion compared to the Model group (Figure 4E). Notably, IFN-γ levels in the HT042 group reached 99.6% of the normal control (NC) values, representing a near-complete restoration and a two-fold increase relative to the Model group (Figure 4E). These findings indicate that HT042 significantly restored hematological parameters and innate immune function in CYP-induced immunosuppressed mice.

### 2.5. Effects of HT042 on the T-Cell-Related Gene of Mice with CYP-Induced Immunosuppression 

**Figure 5:**
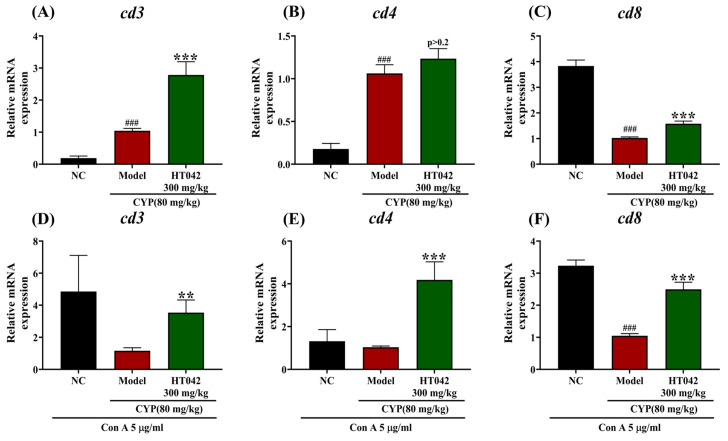
Effects of HT042 on T-cell-related genes in mice with CYP-induced immunosuppression. Mice in the HT042 group received oral administration of HT042 (300 mg/kg/day) for 10 days prior to CYP treatment. Distilled water was used as a vehicle. CYP (80 mg/kg) was injected intraperitoneally to induce immunosuppression. The mRNA expression levels of *Cd3*, *Cd4*, and *Cd8* in splenocytes were analyzed by qRT-PCR. (**A**–**C**) Expression levels at the time of sacrifice. (**D**–**F**) Expression levels in cells cultured for 3 days under infection-like conditions induced by Con A stimulation. Data are presented as mean ± SEM. **, *p* < 0.01 and ***, *p* < 0.001 represents the difference between Model and HT042 groups; ###, *p* < 0.001 represents the difference between the NC and Model groups. NC: normal control; CYP: cyclophosphamide.

Primary splenocytes were isolated from immunosuppressed mice at the time of sacrifice to assess T cell activation at the gene expression level. To simulate an infection-like environment, cells were stimulated with concanavalin A (Con A). The mRNA expression levels of CD3, CD4, and CD8 were significantly upregulated in the HT042 group compared to the Model group (Figure 5A–C). Among these, CD3 and CD8 expressions showed statistically significant increases (Figure 5A,C), whereas the increase in CD4 expression increased did not reach statistical significance (*p* > 0.2) (Figure 5B). In comparison to the normal control (NC), the CYP-treated group exhibited an increased expression of CD3 and CD4. Upon Con A stimulation, the NC group displayed higher expression levels than the Model group, with CD8 showing a significant difference (Figure 5D,E). Notably, HT042 treatment significantly upregulated CD3 (*p <* 0.01), CD4, and CD8 expression (*p*< 0.001) under Con A stimulation. Although the elevation of CD4^+^ T cell activation in the HT042 group was not statistically significant under non-stimulated conditions, it was significantly enhanced upon Con A stimulation (*p* < 0.001), suggesting that HT042 facilitates the restoration of T cell responsiveness under immune-stimulatory conditions.

### 2.6. Effects of HT042 on CD4^+^ Th Subsets of Mice with CYP-Induced Immunosuppression 

**Figure 6:**
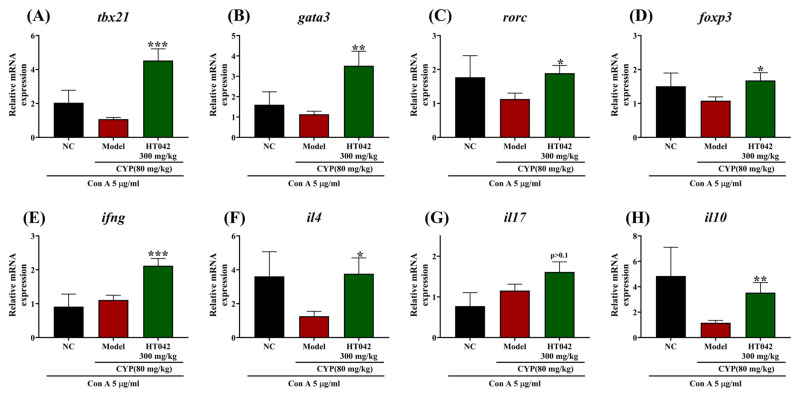
Effect of HT042 on transcription factors of CD4^+^ T helper cell subsets and related cytokines. Mice in the HT042 group received oral administration of HT042 (300 mg/kg/day) for 10 days prior to CYP treatment. Distilled water was used as a vehicle. CYP (80 mg/kg) was injected intraperitoneally to induce immunosuppression. The mRNA expression levels of transcription factors and corresponding cytokines were analyzed by qRT-PCR. (**A**–**D**) Expression levels of transcription factors for CD4^+^ T helper cell subsets; (**E**–**H**) expression levels of representative cytokines under Con A stimulation. The mRNA of transcription factor–cytokine pairs are as follows: Th1 (tbx-ifng), Th2 (gata3-il4), Th17 (rorc-il17), and Treg (foxp3-il10). All data are shown as mean ± SEM. *, *p* < 0.05, **, *p* < 0.01 and ***, *p* < 0.001 represent differences between Model and HT042 groups. NC: normal control; CYP: cyclophosphamide.

**Figure 7:**
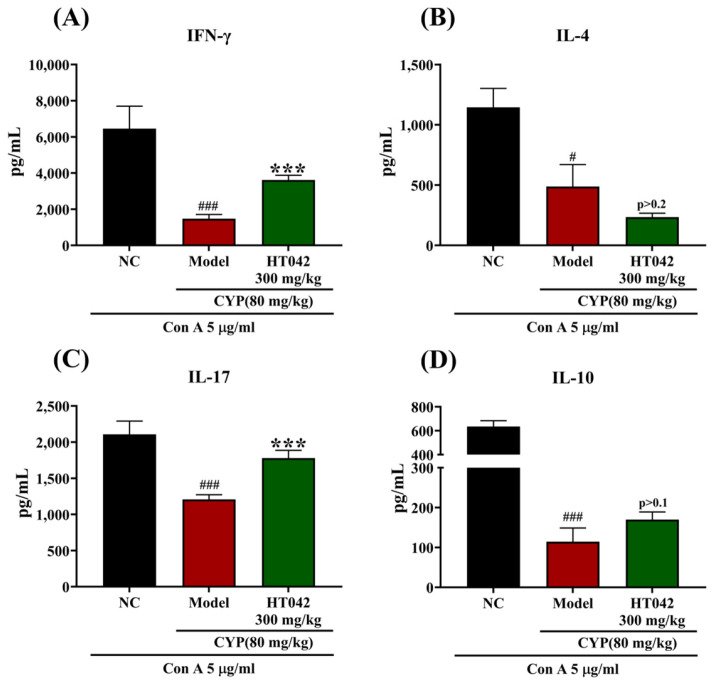
Effects of HT042 on CD4^+^ Th cell cytokines in mice with CYP-induced immunosuppression. Mice in the HT042 group received oral administration of HT042 (300 mg/kg/day) for 10 days prior to CYP treatment. Distilled water was used as a vehicle. CYP (80 mg/kg) was injected intraperitoneally to induce immunosuppression. The cytokine productions under Con A stimulation were measured in cell supernatants by ELISA. (**A**) IFN-γ, (**B**) IL-4, (**C**) IL-17, and (**D**) IL-10. All data are shown as mean ± SEM. ***, *p* < 0.001 represent differences between Model and HT042 groups; #, *p* < 0.05; and ###, *p* < 0.001 represent differences between NC and Model groups. NC: normal control; CYP: cyclophosphamide.

Given the enhanced activity of CD4^+^ T cells under HT042 treatment in an infection-like environment, we assessed the expression of transcription factors and signature cytokines associated with CD4^+^ T helper (Th) cell subsets following Con A stimulation. Under Con-A-stimulated conditions, HT042 treatment significantly upregulated the expression of the transcription factors T-bet (Th1), GATA3 (Th2), RORγt (Th17), and Foxp3 (Treg) compared to the Model group (Figure 6A–D). Correspondingly, HT042 significantly enhanced the mRNA levels of IFN-γ, IL-4, and IL-10, but not IL-17. Although IL-17 expression was approximately twofold higher than in the Model group, this difference did not reach statistical significance (*p* > 0.1) (Figure 6G). These alterations were consistent with the protein levels of representative cytokines measured in culture supernatants (Figure 7). CYP administration significantly suppressed the production of IFN-γ, IL-4, IL-17, and IL-10 in the Model group. HT042 treatment markedly restored IFN-γ and IL-17 levels to near-normal levels (*p* < 0.001), whereas IL-4 levels were further reduced. Although IL-10 levels were increased by HT042 compared to the Model group, this change was not statistically significant (*p* = 0.1).

### 2.7. Effects of HT042 on NO and Cytokine Production in RAW 264.7 Macrophages

**Figure 8:**
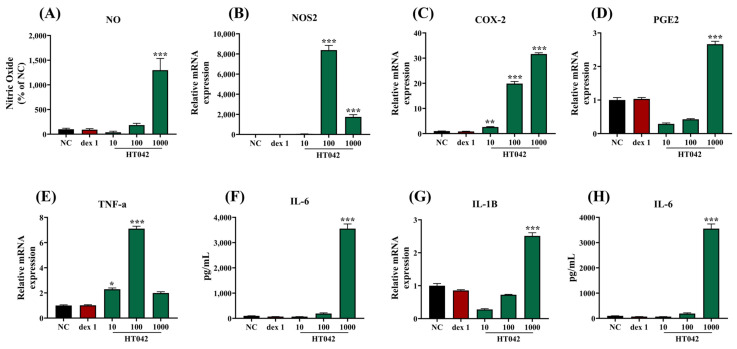
Effects of HT042 on NO production and inflammatory mediators in RAW 264.7 macrophages: (**A**) NO production, (**B**–**G**) mRNA expressions of NOS2, COX-2, PGE2, TNF- α, IL-6 and IL-1β (**H**) protein secretion of IL-6 from RAW 264.7. Gene expression was analyzed using qRT-PCR, and protein levels were quantified via ELISA. All data are exhibited as mean ± SEM. *, *p* < 0.05; **, *p* < 0.01; and ***, *p* < 0.001 represent significance compared to the NC by one-way ANOVA followed by Dunnett’s multiple comparisons test. NC: normal control; NOS2: nitric oxide synthase 2, COX-2: cyclooxygenase 2; TNF: tumor necrosis factor.

NO production was measured in RAW 264.7 cells to assess the proinflammatory effects of HT042. The mRNA expressions of NOS2, PGE2, TNF- α, and COX-2, and inflammatory cytokines IL-6 and IL-1β were analyzed to identify the factors that HT042 regulates (Figure 8). HT042 treatment induced a dose-dependent rise in NO production, in parallel with the upregulation of the mRNA levels of NOS2, COX-2, PGE2, and TNF-α. HT042 treatment also led to dose-dependent augmentation in mRNA expression of inflammatory cytokines, aligning with the elevated protein level of IL-6.

## 3. Discussion

HT042 attenuated CYP-induced weight loss and restored hematological parameters including WBC, RBC, hemoglobin, and platelet counts in immunosuppressed rats, while enhancing NK cell activity and splenocyte proliferation. Ex vivo HT042-treated splenocytes stimulated with ConA showed increased expression of T cell markers (CD3, CD4, CD8), which was paralleled by the increased expression of Th subset-associated transcription factors (GATA3,T-bet, RORγt, Foxp3), along with elevated mRNA and serum levels of their respective cytokines (IL-4, IFN-γ, IL-17, IL-10). In RAW 264.7 macrophages, HT042 enhanced nitric oxide production and increased the expression of NOS2, COX-2, TNF-α, IL-6, and IL-1β. These results indicate that HT042 enhanced both innate and adaptive immune pathways under immunosuppressive conditions, notably promoting T cell subset activation. The implications of these observations are explored in the discussion below.

CYP, an alkylating chemotherapeutic agent, induces cytotoxicity by forming interstrand DNA cross-links that disrupt replication and lead to apoptotic or necrotic cell death. It preferentially targets highly proliferative S-phase cells, including cancer cells, hematopoietic cells, and intestinal epithelial cells [18]. High-dose CYP is associated with weight loss, leukopenia, and the atrophy of immune organs. The observed weight loss is primarily attributed to CYP-induced cytotoxicity and the depletion of mononuclear phagocytes, both of which contribute to immune dysfunction [19,20]. This immunosuppressive state is characterized by reduced NK cell cytotoxicity, impaired phagocytic activity, and attenuated CD4^+^ T cell activation—effects that are further worsened by the inhibition of leptin-mediated signaling pathways [21]. In this study, HT042 alleviated CYP-induced body-weight loss and hematological suppression. This effect was further supported by in vitro data demonstrating the increased proliferative capacity of splenocytes isolated from CYP-treated mice. Extracts of *Astragalus mongholicus* Bunge and their active constituents enhance splenocyte proliferation and promote peripheral blood cell colony formation in immunosuppressed mouse models [22,23]. These effects may involve the inhibition of mitochondrial apoptosis and the activation of immune-related signaling cascades such as MyD88–NF-κB and MAPK [24]. In addition, extracts and polysaccharides derived from *Eleutherococcus senticosus* (Rupr. & Maxim.) Maxim. have demonstrated strong mitogenic activity in murine splenocytes [25,26]. Taken together, these findings support the role of HT042 in restoring immune function by preventing CYP-induced hematopoietic cell apoptosis. Notably, the immunomodulatory effects of *Astragalus mongholicus* and its polysaccharides have been primarily linked to innate immune mechanisms, especially through NF-κB and MAPK signaling activated by Toll-like receptors such as TLR3 and TLR4. However, prior studies on HT042 suggest the involvement of additional pathways. HT042 has been shown to restore IGF-1 secretion suppressed by immunosuppressants, upregulate the anti-apoptotic protein Bcl-2, and inhibit caspase-9 activity [16]. While primarily studied in the context of growth, HT042’s activation of the JAK2/STAT5 pathway may also contribute to immune modulation through shared signaling networks [27]. These actions align with endocrine–immune crosstalk mediated by the growth hormone (GH)–IGF-1 axis, a mechanism not previously highlighted in studies of HT042’s individual components. To confirm the involvement of these pathways, future studies employing Western blotting, pathway-specific inhibitors, or gene knockdown models will be essential.

HT042 administration significantly ameliorated CYP-induced immunosuppression, as evidenced by the restoration of IFN-γ expression and recovery of NK cell activity. NK cells are cytotoxic lymphocytes that respond rapidly during early immune defense without prior sensitization. Interferon-gamma (IFN-γ) is a pivotal cytokine produced during the initial phase of immune response, promoting NK cell activation and cytolytic activity. IFN-γ levels are widely used as a functional marker of NK cell activity. Previous studies have demonstrated that polysaccharides extracted from Astragalus mongholicus Bunge and its active constituent astragaloside III enhance NK cell-mediated immunity by upregulating the IFN-γ production and activating NK cell receptors such as NKp44 and NKG2D [9,28]. In parallel, *Eleutherococcus senticosus* (Rupr. & Maxim.) Maxim. has been reported to augment IFN-γ secretion and cytotoxicity against murine T cell lymphoma cells [26,29]. Together, these findings suggest that HT042 activates NK cell cytotoxic function and contributes to the restoration of innate immune competence under immunosuppressive conditions.

Under CYP-induced immunosuppression, HT042 significantly promoted T cell activation, as indicated by increased CD3, CD4, and CD8 mRNA expression upon Con-A-mediated TCR stimulation. CD3 is expressed on all T cells and associates with the TCR to form the TCR–CD3 complex during antigen–MHC recognition [30]. CD4 and CD8, co-receptors on helper and cytotoxic T cells, respectively, enhance TCR specificity and amplify downstream signaling via ITAM (immunoreceptor tyrosine-based activation motif) phosphorylation within the CD3 complex [31,32]. Our results suggest that HT042 facilitates T cell proliferation and functional activation, with notable effects on the restoration of CD8^+^ cytotoxic T lymphocytes. In assessing the effects of HT042 on CD4^+^ T cell subset differentiation, we observed that HT042 treatment significantly upregulated subset-specific transcription factors, namely T-bet (Th1), GATA3 (Th2), RORγt (Th17), and Foxp3 (Treg), along with the corresponding cytokines IFN-γ, IL-4, IL-17, and IL-10. All cytokines except IL-4 were also elevated at the protein level. These results indicate that HT042 preferentially promotes Th1 and Th17 differentiation. Th1 differentiation is primarily driven by IFN-γ and IL-12 via STAT1 and STAT4 activation, which promotes further IFN-γ production, macrophage activation, and enhanced CD8^+^ T cell responses. Similarly, IL-6 activates the STAT3 pathway, leading to Th17 differentiation, which facilitates neutrophil recruitment and supports mucosal immunity [33,34]. Given that HT042 increased IFN-γ levels in CYP-treated mice and elevated IL-6 and TNF-α production in RAW 264.7 macrophages, HT042 may activate T cells, at least in part through crosstalk with macrophages and NK cells. Further validation of these pathways will require experiments using Western blotting, pharmacological inhibition, or gene silencing approaches.

Notably, serum IL-4 levels remained unchanged in the HT042-treated group, despite an increase in IL-4 mRNA expression. This discrepancy may indicate the post-transcriptional regulation of Th2 cytokines, potentially attenuating allergic inflammation. Formononetin and astragaloside IV, active constituents of *Astragalus mongholicus*, have been shown to suppress Th2 cytokines such as IL-4 and reduce eosinophilic infiltration in allergic asthma models [35]. These results indicate that HT042 not only facilitates Th1- and Th17-mediated immune activation but also supports immune homeostasis and alleviates hypersensitivity through Th2 regulation. Although our gene expression data indicates the potential differentiation of T helper cell subsets, no functional validation was conducted. Thus, these results should be interpreted as indicative rather than conclusive. Future studies employing flow cytometry (FACS) analysis and intracellular cytokine staining will be essential to confirm these findings. Furthermore, previous studies have shown that astragalus polysaccharide compound (ASPC) not only promotes T and B cell proliferation but also markedly enhances the production of adaptive immune mediators including immunoglobulins (IgA, IgG, IgM), cytokines, and complement components (C3, C4) in mice with CYP-induced immunosuppression [36,37]. APS and *Eleutherococcus senticosus* (Rupr. & Maxim.) Maxim. extract have also been reported to enhance polyclonal IgM synthesis and increase the number of IgM-producing plaque-forming cells [9,25]. Taken together, these findings imply that HT042 may facilitate a dual enhancement of innate and adaptive immune functions.

This study demonstrated the immunomodulatory potential of HT042, a functional ingredient known to support growth in children, using a CYP-induced-immunosuppression mouse model and in vitro assays. While the findings provide important insights, several limitations should be acknowledged. First, this study employed a single immunosuppressive model which, although widely used, may not fully capture the diverse immunosuppressive mechanisms associated with other pharmacological agents (e.g., methotrexate, dexamethasone) or pathological conditions (e.g., malnutrition, chronic infection). Incorporating diverse models would enhance the generalizability and validity of the results. Second, immune restoration was evaluated over a relatively short period. Thus, the long-term immunological effects of HT042—including its impact on adaptive immunity, tolerance, toxicity, and safety under chronic or repeated administration—remain to be clarified. Considering that the potential risk of immune-enhancing agents triggering autoimmune or hypersensitivity reactions, thorough evaluations of safety are essential in further studies. Finally, although this study examined key immunological indicators such as cytokines, cell surface markers, and transcription factors, it did not explore how these molecular changes translate into downstream immune functions such as cytotoxicity, antibody production, or antigen presentation. Further mechanistic investigations employing gene knockdown or knockout models are needed to elucidate the molecular pathways underlying HT042-mediated immune modulation.

## 4. Materials and Methods

### 4.1. Sample Preparation and HPLC Analysis

HT042 was provided by NEUMED Inc., (Seoul, Republic of Korea). HT042 was manufactured and underwent quality control following the regulatory standards established by the MFDS (Ministry of Food and Drug Safety). HT042 was prepared by mixing extracts of *Astragalus mongholicus* Bunge, *Eleutherococcus senticosus* (Rupr. & Maxim.) Maxim., and *Phlomis umbrosa* (Turcz.) Kamelin & Makhm in a fixed ratio of 26.5:31.2:42.3. The composition of HT042 and estimated doses of individual components were shown in Table 1. Each sample underwent chromatographic analysis using an Agilent 1260 Infinity System (Agilent, Santa Clara, CA, USA). A reverse-phase ZORBAX Eclipse Plus C18 column (4.6 × 250 mm, 5 µm, Agilnet) was utilized. The mobile phase was composed of 0.5% phosphoric acid (A) and acetonitrile (B). For the simultaneous analysis of eleutheroside E and shanzhiside methyl ester, the gradient was as follows: 0–20 min, 5–17% B; 20–30 min, 17–22% B; 30–40 min, 22–30% B; 40–43 min, 30–100% B; 43–45 min, 100% B; 45–47 min, 100–5% B; and 47–50 min, 5% B. For the analysis of formononetin, the gradient was as follows: 0–15 min, 35% B, 15–25 min 35–65% B; 25–28 min, 65–35% B; 28–30 min, 35% B. The elution was used and kept at 40 °C. The flow rate was 1.0 mL/min. Eleutheroside E, shanzhiside methyl ester, and formononetin were detected at 210, 235, and 245 nm, respectively. Standardized HT042 comprised 0.36% eleutheroside E, 0.15% shanzhiside methyl ester, and 0.008% formononetin. 

### 4.2. Animals

Male BALB/c mice (*n* = 54), at six weeks of age, were purchased from NARA BIO Co., Ltd. (Pyeongtaek, Republic of Korea). Upon arrival, the mice were housed under conventional laboratory conditions within an animal chamber, with a controlled environment maintained with a humidity level of 55 ± 5%, a 12 h light/dark cycle, and a temperature of 22 ± 1 °C. Mice had unrestricted access to standard rodent diet and distilled water (ad libitum). Prior to commencing in vivo efficacy testing, the mice underwent a 7-day acclimatization period to adapt to the facility conditions. All procedures and protocols involving animal handling and care were approved by the Institutional Animal Care and Use Committee (IACUC) of NEUMED, Inc. (approval number: KISTEM-IACUC-2024-004).

### 4.3. Development of the Immune-Suppressed Mouse Model

After a 7-day acclimation period, mice (body weight of 21.8 ± 0.2 g) were randomly assigned into 3 groups, *n* = 18 per group: normal control (NC), cyclophosphamide (CYP, Sigma, New York, NY, USA) with distilled water (Model), and CYP with HT042 (HT042). Sample size was calculated according to following formula = 2 SD^2^ (Z^α/2^ + Z^β^) 2/d^2^. Where standard deviation (SD) was derived from pilot studies, and d represents the effect size, defined as the difference between mean values from previous studies [38]. To prevent aggressive interactions that could influence immune responses, each pair of mice were housed in isolator cages with physical barriers, allowing two mice per cage without direct interaction. Mice in the NC and Model groups received distilled water at a dose of 10 mL/kg body weight, whereas the HT042 group were given HT042 at a dose of 300 mg/kg. The HT042 dosage was determined based on previously established protocols from both preclinical and clinical research [39,40]. The dosage applied in the clinical trial was calculated using a standard human-to-mouse dose conversion formula [41]. All treatments were administered orally, once daily at the same time each morning (9:00 AM) from Day 1 through Day 13. On Day 11, one hour following the administration of distilled water or HT042, all mice in the Model and HT042 groups received an intraperitoneal injection of CYP at a dose of 80 mg/kg to establish the immune-suppressed condition. Body weights were recorded daily from Day 11 to Day 14. In accordance with animal ethics regulations, mice that exhibited a weight loss of more than 20% were to be euthanized. No exclusions occurred during the experiment. On Day 14, all mice were anesthetized, blood samples were collected via cardiac puncture. After blood collection, all animals were sacrificed following institutional ethical guidelines for animal research.

### 4.4. Hematological Analysis and NK Cell Activity Measurement

Whole blood was collected from each mouse and stored in 3 mL K2-EDTA vacutainer tubes (Becton, Dickinson and Company, Franklin Lakes, NJ, USA). Blood samples were analyzed by SCL Healthcare (Seoul, Korea), a specialized clinical laboratory, to assess various hematological parameters, such as white blood cell (WBCs), red blood cell (RBCs), hemoglobin, and platelet levels. Additionally, approximately 100 μL of heparinized whole blood was used to evaluate natural killer (NK) cell activity by measuring interferon-gamma (IFN-γ) secretion levels. This assay was performed using the Murine NK Cell Activity ELISA Kit (NKMAX Bio, Seongnam-si, Korea) according to the manufacturer’s protocol.

### 4.5. Primary Splenocyte Proliferation Analysis

Primary splenocytes were isolated from the spleens of seven-week-old C57BL/6N mice. Following isolation, the cells were counted and seeded at a density of 2×10⁵ cells per 100 μL of medium into 96-well plate. HT042 was then administered at two experimental concentrations: 10 μg/mL and 100 μg/mL. Control wells received an equivalent volume of distilled water in place of HT042. To model immunosuppressive condition, CYP was applied in combination with HT042. In these wells, 1.6 μg/mL of CYP was added 2 h after the initial HT042 treatment, following the same concentration protocol for HT042. All experimental groups were then incubated for a total of 22 h under 37 °C in a humidified atmosphere containing 5% CO_2_. After incubation, cell viability was assessed by adding 10 μL of Cell Counting Kit-8 (CCK-8; Dojindo Laboratories, Japan) to each well. The cells were then incubated with CCK-8 for an additional 2 h at 37 °C, and the optical density (OD) was analyzed at 450 nm.

### 4.6. Primary Splenocyte Culture Under Concanavalin A Treatment

After sacrifice, spleens were harvested from all groups of mice and dissociated into single-cell suspensions. The cells, density of 2 × 10^6^ cells/mL per well, were seeded in a 24-well plate. Each well was treated with Concanavalin A (Con A; Sigma, United States) at a final concentration of 5 μg/mL to stimulate the cells. Following a 72-h incubation, both cells and culture supernatants were collected and centrifuged to separate cell pellets from the supernatants. The resulting cell pellets and supernatants were stored at −70 °C until further analysis.

### 4.7. Real-Time Reverse Transcription Polymerase Chain Reaction (RT-PCR)

Total RNA was isolated from spleen tissues utilizing the AccuPrep^®^ Universal RNA Extraction Kit (Bioneer, Daejeon, Korea). The cDNA was generated from 1 µg of total RNA employing a High-Capacity cDNA Reverse Transcription Kit (Thermo Fisher, Waltham, MA, USA), according to the protocol provided by the manufacturer. The primers used for quantifying gene expression levels, along with their specific sequences, are detailed in Table 2 and Table 3.

### 4.8. Cytokine Measurement Using ELISA

Cytokine levels in the cell culture supernatants were quantified using the ELISA Duoset sandwich enzyme-linked immunosorbent assay kit (R&D Systems, Rueil-Malmaison, France) in accordance with the manufacturer’s protocol.

### 4.9. Cell Culture and NO Assay of RAW 264.7 Macrophages

RAW 264.7 cells, provided by the Korean Cell Line Bank (Seoul, Republic of Korea), were cultured in DMEM supplemented with 10% fetal bovine serum and 1% penicillin/streptomycin (Gibco BRL, Carlsbad, CA, USA). RAW 264.7 cells (5.0 × 10^5^ cells/well) were seeded in 96-well plates and incubated with HT042 at concentrations ranging from 10 to 1000 µg/mL for 24 h. NO production was assessed by mixing the supernatant with Griess reagent, followed by measurement of absorbance at 540 nm.

### 4.10. Statistical Analysis

Two-way ANOVA was conducted to evaluate differences in body weight and weight gain across the three groups (NC, Model, and HT042), followed by Dunnett’s post hoc test to compare the treatment group with the Model group. For other efficacy-related parameters, unpaired t-tests were performed to compare the Model and HT042 groups. Additionally, to assess the systemic impact of immunosuppression itself, a separate unpaired *t*-test was conducted between the NC and Model groups. Data are presented as the mean ± standard error of the mean (SEM) from three independent experiments, and statistical significance was defined as *p* < 0.05, *p* < 0.01, or *p* < 0.001. All analyses were conducted using GraphPad Prism software (version 8.4.2, GraphPad Software, Boston, MA, USA).

## 5. Conclusions

In conclusion, this study demonstrates that the Astragalus extract mixture HT042 effectively restores immune competence in mice with CYP-induced immunosuppression and RAW 264.7 macrophages. These findings collectively suggest that HT042 facilitates a coordinated enhancement of innate and adaptive immunity, primarily via T cell modulation. Furthermore, its immunostimulatory effects appear to be mediated, at least in part, through the activation of macrophage–NK cell crosstalk and the amplification of downstream pro-inflammatory signaling cascades. To fully assess its therapeutic potential, further validation of HT042 is required in advanced experimental models and under diverse immunosuppressive conditions, including long-term administration. Future studies should clarify its dose–response characteristics, determine the mechanisms of action—particularly those involving the GH–IGF-1 axis, and undertake a comprehensive evaluation of risks Lastly, although HT042 has demonstrated safety in short-term studies [42], its long-term safety profile remains to be fully established. Comprehensive evaluation of risks including autoimmune activation, organ-specific toxicity, and immunological tolerance will be essential to establish a reliable basis for clinical translation.

## Figures and Tables

**Table 1:** HT042 composition and estimated doses of individual herbal components.

Botanical Name	Ratio (%)	Estimated Dose (mg/kg)
*Astragalus mongholicus* Bunge	26.5	79.5
*Eleutherococcus senticosus* (Rupr. & Maxim.) Maxim.	31.2	93.6
*Phlomis umbrosa* (Turcz.) Kamelin & Makhm	42.3	126.9

**Table 2:** Primer sequences for splenocytes of mice with CYP-induced immunosuppression.

Gene	Primer	Sequence
CD3	Forward	GCTCCAGGATTTCTCGGAAGTC
	Reverse	ATGGCTACTGCTGTCAGGTCCA
CD4	Forward	GTTCAGGACAGCGACTTCTGGA
	Reverse	GAAGGAGAACTCCGCTGACTCT
CD8α	Forward	ACTACCAAGCCAGTGCTGCGAA
	Reverse	ATCACAGGCGAAGTCCAATCCG
T-bet	Forward	CCACCTGTTGTGGTCCAAGTTC
	Reverse	CCACAAACATCCTGTAATGGCTTG
GATA3	Forward	CCTCTGGAGGAGGAACGCTAAT
	Reverse	GTTTCGGGTCTGGATGCCTTCT
RORγt	Forward	CCGCTGAGAGGGCTTCAC
	Reverse	TGCAGGAGTAGGCCACATTACA
FoxP3	Forward	CCTGGTTGTGAGAAGGTCTTCG
	Reverse	TGCTCCAGAGACTGCACCACTT
IFN-γ	Forward	CAGCAACAGCAAGGCGAAAAAGG
	Reverse	TTTCCGCTTCCTGAGGCTGGAT
IL-4	Forward	ATCATCGGCATTTTGAACGAGGTC
	Reverse	ACCTTGGAAGCCCTACAGACGA
IL-17	Forward	CAGACTACCTCAACCGTTCCAC
	Reverse	TCCAGCTTTCCCTCCGCATTGA
IL-10	Forward	CGGGAAGACAATAACTGCACCC
	Reverse	CGGTTAGCAGTATGTTGTCCAGC
β-actin	Forward	TGCTGTCCCTGTATGCCTCTG
	Reverse	TGATGTCACGCACGATTTCC

CD: Cluster of differentiation, IL: interleukin.

**Table 3:** Primer sequences for RAW 264.7 cells.

Gene	Primer	Sequence
GAPDH	Forward	CTTGTGACAAAGTGGACATTGTT
	Reverse	TGACCAGCTTCCCATTCTC
TNF-α	Forward	GAGAAGTTCCCAAATGGCCT
	Reverse	AGCCACTCCAGCTGCTCCT
COX-2	Forward	ATCCATGTCAAAACCGTGGG
	Reverse	TTGGGGTGGGCTTCAGCAG
NOS2	Forward	ACCAAGATGGCCTGGAGGAA
	Reverse	CCGACCTGATGTTGCCATTG
PGE2	Forward	CTGGTAACGGAATTGGTGC
	Reverse	TGGCCAGACTAAAGAAGGTC
IL-6	Forward	CACTTCACAAGTCGGAGGCT
	Reverse	CAAGTGCATCATCGTTGTTC
IL-1B	Forward	CCAGCTTCAAATCTCGCAGC
	Reverse	GTGCTCATGTCCTCATCCTGG

GAPDH: glyceraldehyde-3-phosphate dehydrogenase, TNF: tumor necrosis factor; NOS2: nitric oxide synthase2, COX-2: cyclooxygenase 2, PGE2: prostaglandin E2, IL: interleukin.

## Data Availability

All data are contained within the article.

## Abbreviations

The following abbreviations are used in this manuscript:

NK	Natural killer
GH	Growth hormone
IGF-1	Insulin-like growth factor-1
CYP	Cyclophosphamide
WBC	White blood cell
RBC	Red blood cell
IFN	Interferon
GAPDH	Glyceraldehyde-3-phosphate dehydrogenase
TNF	Tumor necrosis factor
NOS2	Nitric oxide synthase2
COX-2	Cyclooxygenase 2
PGE2	Prostaglandin E2
IL	Interleukin.
CD	Cluster of differentiation
NO	Nitric oxide
Con A	Concanavalin A
Th	T helper
